# Supplementary material for: Nutritional Supplementation Reduces Lesion Size and Neuroinflammation in a Sex-Dependent Manner in a Mouse Model of Perinatal Hypoxic-Ischemic Brain Injury
Source: Nutrients. 2021 Dec 30;14(1):176. doi: 10.3390/nu14010176 (PMC8747710; doi:10.3390/nu14010176)
Supplement: Supplementary file 1 [file nutrients-14-00176-s001.zip › nutrients-1490950 Supplementary.pdf]

Supplementary Materials

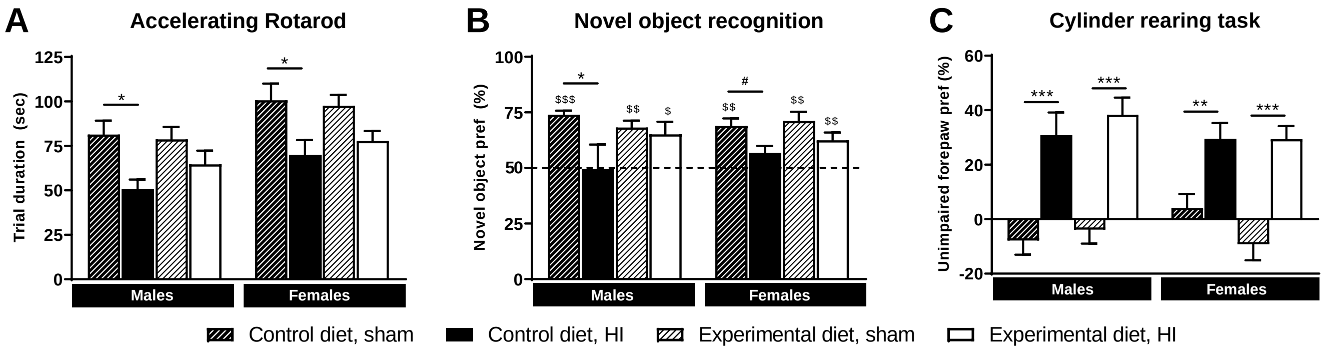

Supplementary Figure S1. Behavioral tasks separated by sex (**A**, Rotarod: control diet, sham, *n* = 12/13 male/female, control diet, HI, *n* = 11/14, experimental diet, sham, *n* = 12/15, experimental diet, HI, *n* = 14/14, **B**, Novel object recognition: control diet, sham, *n* = 4/6 male/female, control diet, HI, *n* = 4/7, experimental diet, sham, *n* = 7/7, experimental diet, HI, *n* = 6/8, **C**, Cylinder rearing task: control diet, sham, *n* = 12/14 male/female, control diet, HI, *n* = 8/13, experimental diet, sham, *n* = 13/15, experimental diet, HI, *n* = 14/13). P-values are corrected for three comparisons (males and females are analyzed separately). # corrected $p < .10$, * corrected $p < 0.05$, ** corrected $p < 0.01$, *** corrected $p < 0.001$, $ $p < 0.05$ compared to 50%, $$ $p < 0.01$ compared to 50%, $$$ $p < 0.001$ compared to 50%

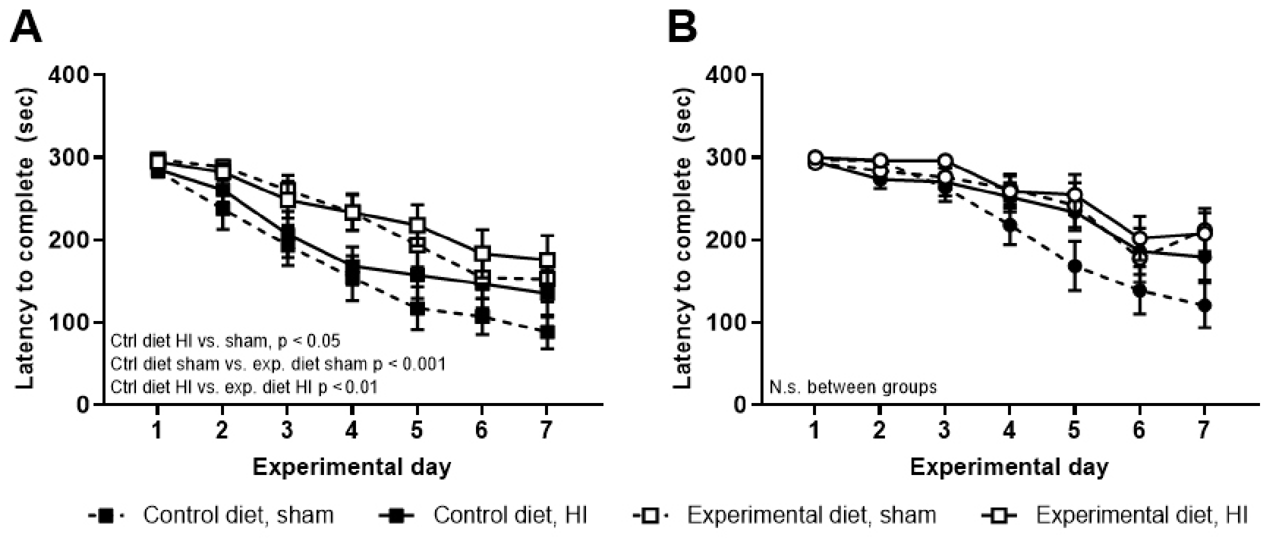

Supplementary Figure S2. Performance on the modified holeboard for males (**A**) and females (**B**). Male mice that were fed the experimental diet (both HI and sham groups) took a longer time to collect all three baits over the 7 testing days. There was no difference between HI and sham in the experimental diet group, whereas this difference was found for male mice that were fed the control diet. There were no effects of HI or diet found for females. Control diet, sham, n = 12/12 male/female, control diet, HI, n = 11/12, experimental diet, sham, n = 13/10, experimental diet, HI, n = 14/11

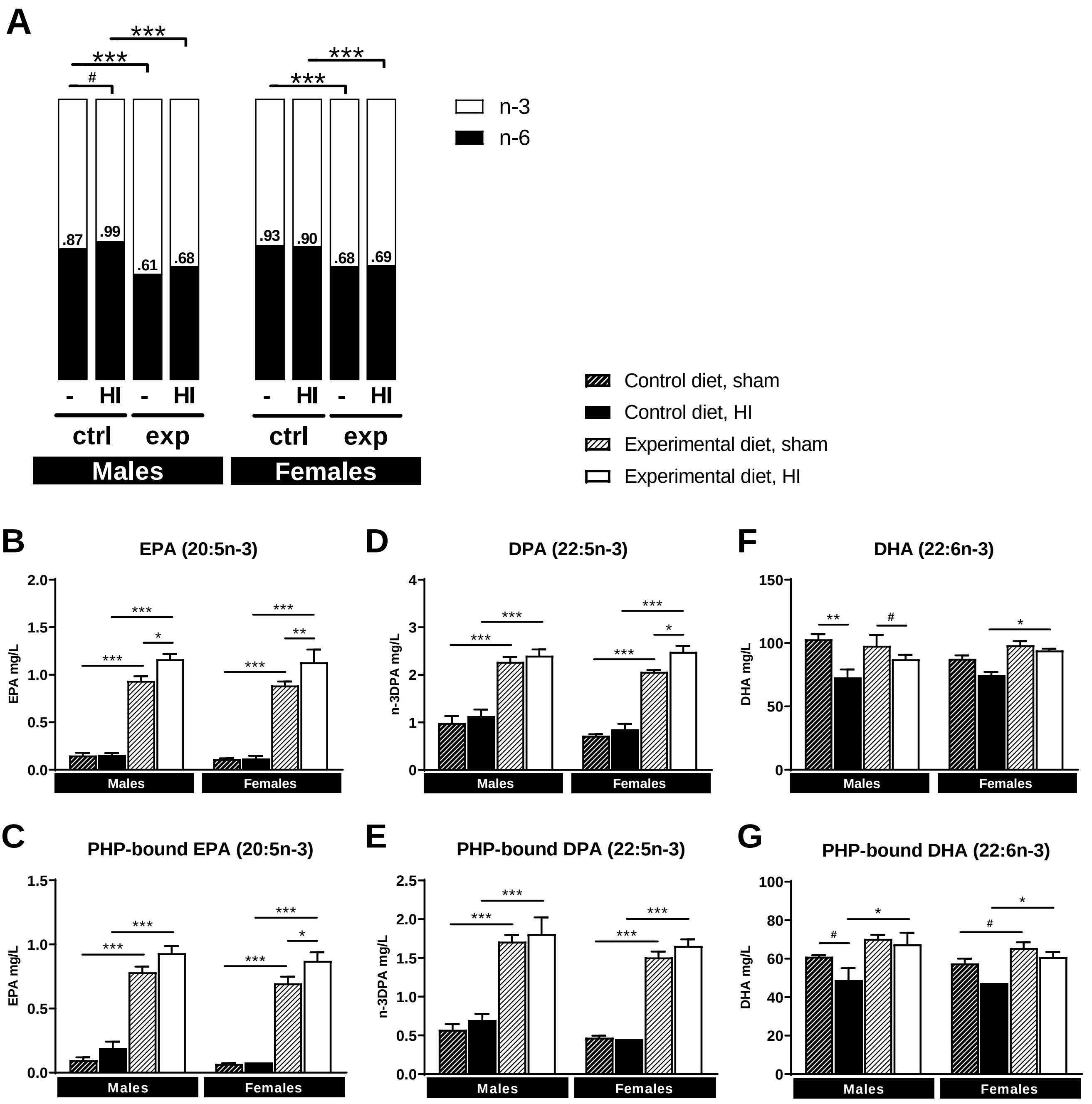

Supplementary Figure S3. Total fatty acid levels (**A**, **B**, **D**, **F**) and phospholipid-bound fatty acid levels (**C**, **E**, **G**) as mg/L in the ipsilateral hemisphere, separated by sex. (**A**, **B**, **D**, **F**): control diet, sham, n = 7/6 male/female, control diet, HI, n = 5/5, experimental diet, sham, n = 6/7, experimental diet, HI, n = 8/6, (**C**, **E**, **G**): control diet, sham, n = 6/6 male/female, control diet, HI, n = 5/1, experimental diet, sham, n = 6/7, experimental diet, HI, n = 3/5, # corrected $p < 0.10$, * corrected $p < 0.05$, ** corrected $p < 0.01$, *** corrected $p < 0.001$

Supplementary Table S1. Fatty acid composition in mg/L in the ipsilateral hemisphere of the cerebrum.

		Control diet		Experimental diet		*p*		
		**Sham**	**HI**	**Sham**	**HI**	**HI**	**Diet**	**HIxD**
n-3 FAs	Total	96.14 ± 4.74	76.58 ± 3.45	105.89 ± 2.58	95.81 ± 2.13	< 0.05	< 0.10	-
18:3n-3	ALA	2.34 ± 0.25	1.87 ± 0.18	2.14 ± 0.09	1.76 ± 0.10	< 0.10	-	-
20:5n-3	EPA	0.13 ± 0.02	0.14 ± 0.02	0.91 ± 0.03	1.15 ± 0.07	< 0.001	< 0.001	< 0.01
22:5n-3	DPA	0.86 ± 0.08	0.99 ± 0.10	2.17 ± 0.06	2.44 ± 0.09	< 0.05	< 0.001	-
22:6n-3	DHA	92.81 ± 4.52	73.56 ± 3.30	100.66 ± 2.59	90.45 ± 2.17	< 0.05	< 0.10	-
n-6 FAs	Total	86.36 ± 5.11	71.64 ± 2.48	67.75 ± 1.30	65.57 ± 1.51	-	< 0.001	< 0.05
18:2n-6	LA	2.83 ± 0.17	2.34 ± 0.13	2.68 ± 0.10	2.52 ± 0.12	-	-	-
20:2n-6	-	1.31 ± 0.09	1.09 ± 0.06	1.18 ± 0.05	1.04 ± 0.06	-	-	-
20:3n-6	DGLA	2.14 ± 0.23	1.80 ± 0.14	2.80 ± 0.05	2.69 ± 0.06	-	< 0.01	-
20:4n-6	ARA	59.72 ± 3.62	48.94 ± 1.66	46.49 ± 1.14	45.24 ± 1.47	-	< 0.001	< 0.05
22:4n-6	DTA	17.25 ± 1.24	15.18 ± 0.59	12.15 ± 0.43	11.75 ± 0.28	-	< 0.001	-
22:5n-6	DPA	2.68 ± 0.26	1.95 ± 0.12	2.12 ± 0.04	1.97 ± 0.05	-	< 0.05	< 0.05
24:2n-6	-	0.44 ± 0.12	0.34 ± 0.09	0.34 ± 0.07	0.36 ± 0.08	-	-	-
n-9 FAs	Total	125.16 ± 9.96	101.20 ± 6.99	122.94 ± 2.81	110.15 ± 3.84	-	-	-
18:1n-9	Oleic/Elaidic	105.13 ± 7.85	85.26 ± 5.35	104.89 ± 2.22	95.27 ± 2.81	-	-	-
20:1n-9	Gondoic	9.20 ± 1.02	7.49 ± 0.77	8.40 ± 0.33	7.11 ± 0.46	-	-	-
22:1n-9	Erucic	1.04 ± 0.11	0.84 ± 0.09	0.96 ± 0.04	0.79 ± 0.05	-	-	-
24:1n-9	Nervonic	9.79 ± 1.15	7.62 ± 0.82	8.68 ± 0.43	6.98 ± 0.64	-	-	-
Saturated FAs		299.13 ± 17.21	242.38 ± 8.87	285.34 ± 4.66	268.50 ± 5.05	-	-	< 0.05

Mean ± standard error of fatty acids in mg/L. FAs = fatty acids, ALA = alpha linolenic acid, SDA = stearidonic acid, EPA = eicosapentaenoic acid, DPA = docosapentaenoic acid, DHA = docosahexaenoic acid, LA = linolenic acid, GLA = gamma linoleic acid, DGLA = dihomo-gamma linolenic acid, ARA = arachidonic acid, DTA = docosatetraenoic acid, HIxD = interaction effect HI x diet, - no significant effects ($p > 0.10$). Control diet: sham, $n = 10$, HI, $n = 12$, experimental diet: sham, $n = 14$, HI, $n = 13$

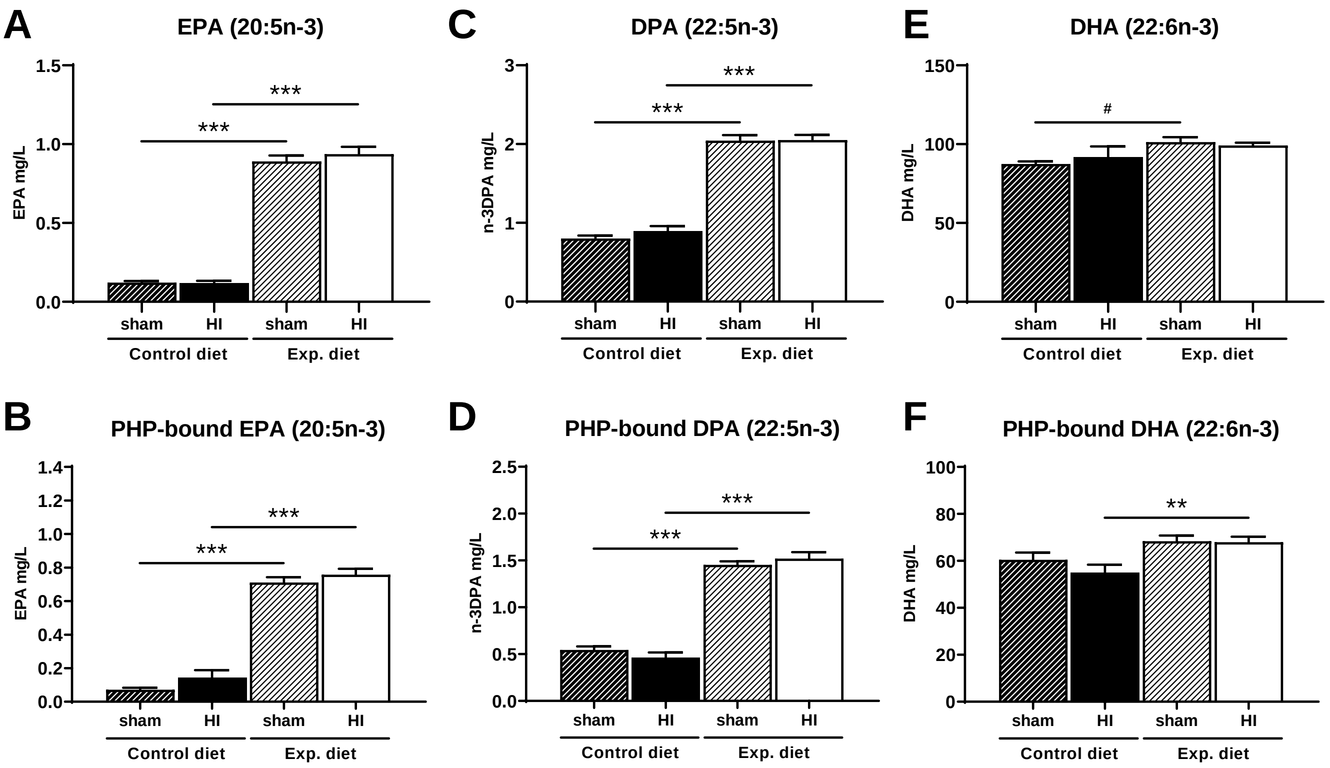

Supplementary Figure S4. Total n-3 fatty acid levels (**A**, **C**, **E**) and phospholipid-bound fatty acid levels (**B**, **D**, **F**) as mg/L in the contralateral hemisphere. (**A**, **C**, **E**): control diet: sham, $n = 12$, HI, $n = 13$, experimental diet: sham, $n = 14$, HI, $n = 16$. (**B**, **D**, **F**): control diet: sham, $n = 11$, HI, $n = 10$, experimental diet: sham, $n = 12$, HI, $n = 16$. PHP = phospholipid, # corrected $p < 0.10$, * corrected $p < 0.05$, ** corrected $p < 0.01$, *** corrected $p < 0.001$

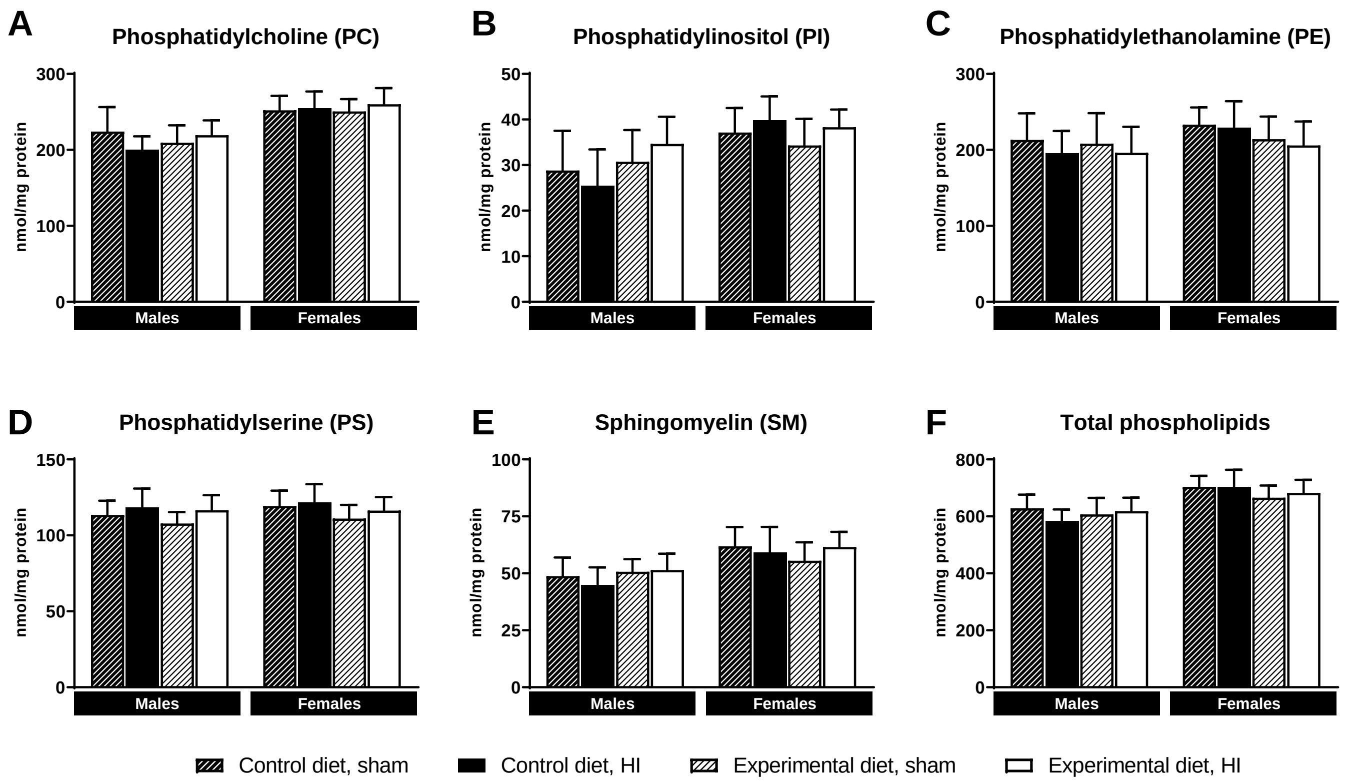

Supplementary Figure S5. Phospholipid levels in nmol/mg protein in the ipsilateral hemisphere of the brain, separated by sex (control diet, sham, *n* = 6/6 male/female, control diet, HI, *n* = 6/7, experimental diet, sham, *n* = 7/7, experimental diet, HI, *n* = 8/8). No differences between groups were found for phospholipid species phosphatidylcholine (**A**), phosphatidylinositol (**B**), phosphatidylserine (**C**), phosphatidylethanolamine (**D**), sphingomyelin (**E**) or the total phospholipids per mg protein (**F**).
